# *L*-Fuzzy Fixed Points Theorems for *L*-Fuzzy Mappings via *β*_*ℱ*_*L*__-Admissible Pair

**DOI:** 10.1155/2014/853032

**Published:** 2014-02-05

**Authors:** Maliha Rashid, Akbar Azam, Nayyar Mehmood

**Affiliations:** ^1^Department of Mathematics, International Islamic University, Sector H-10, Islamabad 44000, Pakistan; ^2^Department of Mathematics, COMSATS Institute of Information Technology, Chack Shahzad, Islamabad 44000, Pakistan

## Abstract

We define the concept of *β*_*ℱ*_*L*__-admissible for a pair of *L*-fuzzy mappings and establish the existence of common *L*-fuzzy fixed point theorem. Our result generalizes some useful results in the literature. We provide an example to support our result.

## 1. Introduction

A large variety of the problems of analysis and applied mathematics relate to finding solutions of nonlinear functional equations which can be formulated in terms of finding the fixed points of nonlinear mappings. Heilpern [1] first introduced the concept of fuzzy mappings and established a fixed point theorem for fuzzy contraction mappings in complete metric linear spaces, which is a fuzzy extension of Banach contraction principle and Nadler's [2] fixed point theorem. Subsequently several other authors [3–17] generalized this result and studied the existence of fixed points and common fixed points of fuzzy mappings satisfying a contractive type condition.

Zadeh published his important paper “*Fuzzy sets*” [18], after that Goguen published the paper “*L*-*Fuzzy sets*” [19]. The concept of *L*-fuzzy sets is a generalization of the concept of fuzzy sets. Fuzzy set is a special case of *L*-fuzzy set when *L* = [0,1]. There are basically two understandings of the meaning of *L*, one is when *L* is a complete lattice equipped with a multiplication ∗ operator satisfying certain conditions as shown in the initial paper [19] and the second understanding of the meaning of *L* is that *L* is a completely distributive complete lattice with an order-reversing involution (see, e.g., [20–22], etc.).

In 2012, Samet et al. [23] introduced the concept of *β*-admissible mapping and established fixed point theorems via *β*-admissible and also showed that these results can be utilized to derive fixed point theorems in partially ordered spaces and coupled fixed point theorems. Moreover, they applied the main results to ordinary differential equations. Afterwards, Asl et al. [24] extended the concept of *β*-admissible for single valued mappings to multivalued mappings. Recently, Mohammadi et al. [25] introduced the concept of *β*-admissible for multivalued mappings which is different from the notion of *β*_∗_-admissible which has been provided in [24] and Azam and Beg [26] obtained a common *α*-fuzzy fixed point of a pair of fuzzy mappings on a complete metric space under a generalized contractive condition for *α*-level sets via Hausdorff metric for fuzzy sets.

In this paper we introduce the concept of *β*_*ℱ*_*L*__-admissible for a pair of *L*-fuzzy mappings and establish the existence of common *L*-fuzzy fixed point theorem. We also have given an example to support our main theorem.

## 2. Preliminaries

Let (*X*, *d*) be a metric space, and denote

 
*CB*(*X*) = {*A* : *A* is nonempty closed and bounded subset of *X*},
 
*C*(*X*) = {*A* : *A* is nonempty compact subset of *X*}.

For *ϵ* > 0 and the sets *A*, *B* ∈ *CB*(*X*) define

(1) $$\begin{array}{c} d\left(x,A\right)=\underset{y\in A}{\inf}\;d\left(x,y\right),\\ d\left(A,B\right)=\underset{x\in A,y\in B}{\inf}d\left(x,y\right),\\ N\left(\epsilon ,A\right)=\left\{x\in X:d\left(x,a\right)<\epsilon ,\;\text{for}\;\;\text{some}\;a\in A\right\},\\ E_{A,B}=\left\{\epsilon >0:A\subseteq N\left(\epsilon ,B\right),B\subseteq N\left(\epsilon ,A\right)\right\}. \end{array}$$

Then the Hausdorff metric *d*_*H*_ on *CB*(*X*) induced by *d* is defined as

(2) $$\begin{array}{c} d_{H}\left(A,B\right)=\inf E_{A,B}. \end{array}$$

**Lemma 1 (see [2])**

Let (*X*, *d*) be a metric space and *A*, *B* ∈ *CB*(*X*); then for each *a* ∈ *A*

(3) $$\begin{array}{c} d\left(a,B\right)\leq H\left(A,B\right). \end{array}$$

**Lemma 2 (see [2])**

Let (*X*, *d*) be a metric space and *A*, *B* ∈ *CB*(*X*); then for each *a* ∈ *A*, *ϵ* > 0, there exists an element *b* ∈ *B* such that

(4) $$\begin{array}{c} d\left(a,b\right)\leq H\left(A,B\right)+\epsilon . \end{array}$$

**Definition 3 (see [19])**

A partially ordered set (*L*, ≾_*L*_) is called

(i) a lattice, if *a*∨*b* ∈ *L*,  *a*∧*b* ∈ *L* for any *a*, *b* ∈ *L*;
(ii) a complete lattice, if ∨*A* ∈ *L*,  ∧*A* ∈ *L* for any *A*⊆*L*;
(iii) distributive if *a*∨(*b*∧*c*) = (*a*∨*b*)∧(*a*∨*c*),  *a*∧(*b*∨*c*) = (*a*∧*b*)∨(*a*∧*c*) for any *a*, *b*, *c* ∈ *L*.

**Definition (see [19])**

Let *L* be a lattice with top element 1_*L*_ and bottom element 0_*L*_ and let *a*, *b* ∈ *L*. Then *b* is called a complement of *a*, if *a*∨*b* = 1_*L*_, and *a*∧*b* = 0_*L*_. If *a* ∈ *L* has a complement element, then it is unique. It is denoted by $\overset{´}{a}$.

**Definition (see [19])**

An *L*-fuzzy set *A* on a nonempty set *X* is a function *A* : *X* → *L*, where *L* is complete distributive lattice with 1_*L*_ and 0_*L*_.

**Remark**

The class of *L*-fuzzy sets is larger than the class of fuzzy sets as an *L*-fuzzy set is a fuzzy set if *L* = [0,1].

The *α*_*L*_-level set of *L*-fuzzy set *A* is denoted by *A*_*α*_*L*__ and is defined as follows:

(5) $$\begin{array}{c} A_{\alpha_{L}}=\left\{x:\alpha_{L}\;{≾}_{L}\;A\left(x\right)\right\}\;\text{if}\;\;\alpha_{L}\in L\setminus \left\{0_{L}\right\},\\ A_{0_{L}}=\mathrm{cl}\left(\left\{x:0_{L}\;{≾}_{L}\;A\left(x\right)\right\}\right). \end{array}$$

Here cl⁡(*B*) denotes the closure of the set *B*.

We denote and define the characteristic function *χ*_*L*_*A*__ of an *L*-fuzzy set *A* as follows:

(6) $$\begin{array}{c} \chi_{L_{A}}:=\{\begin{array}{cc} 0_{L} & \text{if}\;\;x\notin A,\\ 1_{L} & \text{if}\;\;x\in A. \end{array} \end{array}$$

**Definition 7**

Let *X* be an arbitrary set and *Y* a metric space. A mapping *T* is called *L*-fuzzy mapping if *T* is a mapping from *X* into *ℱ*_*L*_(*Y*). An *L*-fuzzy mapping *T* is an *L*-fuzzy subset on *X* × *Y* with membership function *T*(*x*)(*y*). The function *T*(*x*)(*y*) is the grade of membership of *y* in *T*(*x*).

**Definition 8**

Let (*X*, *d*) be a metric space and *S*, *TL*-fuzzy mappings from *X* into *ℱ*_*L*_(*X*). A point *z* ∈ *X* is called an *L*-fuzzy fixed point of *T* if *z* ∈ [*Tz*]_*α*_*L*__, where *α*_*L*_ ∈ *L*∖{0_*L*_}. The point *z* ∈ *X* is called a common *L*-fuzzy fixed point of *S* and *T* if *z* ∈ [*Sz*]_*α*_*L*__∩[*Tz*]_*α*_*L*__.

**Definition (see [23])**

Let *X* be a nonempty set, *T* : *X* → *X*, and *β* : *X* × *X* → [0, *∞*). We say that *T* is *β*-admissible if for all *x*, *y* ∈ *X* we have

(7) $$\begin{array}{c} \beta \left(x,y\right)\geq 1⟹\beta \left(Tx,Ty\right)\geq 1. \end{array}$$

**Definition 10 (see [24])**

Let *X* be a nonempty set, *T* : *X* → 2^*X*^, where 2^*X*^ is a collection of subset of *X*, *β* : *X* × *X* → [0, *∞*) and *β*_∗_ : 2^*X*^ × 2^*X*^ → [0, *∞*). We say that *T* is *β*_∗_-admissible if for all *x*, *y* ∈ *X* we have

(8) $$\begin{array}{c} \beta \left(x,y\right)\geq 1⟹\beta_{*}\left(Tx,Ty\right)\geq 1. \end{array}$$

**Definition 11 (see [25])**

Let *X* be a nonempty set, *T* : *X* → 2^*X*^, where 2^*X*^ is a collection of subset of *X* and *β* : *X* × *X* → [0, *∞*). We say that *T* is *β*-admissible whenever for each *x* ∈ *X* and *y* ∈ *Tx* with *β*(*x*, *y*) ≥ 1 we have *β*(*y*, *z*) ≥ 1 for all *z* ∈ *Ty*.

## 3. Main Result

In this section, we introduce a new concept of *β*_*ℱ*_*L*__-admissible for a pair of *L*-fuzzy mappings and establish the existence of common *L*-fuzzy fixed point theorem.

**Definition**

Let (*X*, *d*) be a metric space, *β* : *X* × *X* → [0, *∞*), and *S*, *TL*-fuzzy mappings from *X* into *ℱ*_*L*_(*X*). The order pair (*S*, *T*) is said to be *β*_*ℱ*_*L*__-admissible if it satisfies the following conditions:

(i) for each *x* ∈ *X* and *y* ∈ [*Sx*]_*α*_*L*_(*x*)_, where *α*_*L*_(*x*) ∈ *L*∖{0_*L*_}, with *β*(*x*, *y*) ≥ 1, we have *β*(*y*, *z*) ≥ 1 for all *z* ∈ [*Ty*]_*α*_*L*_(*y*)_ ≠ *ϕ*, where *α*_*L*_(*y*) ∈ *L*∖{0_*L*_};
(ii) for each *x* ∈ *X* and *y* ∈ [*Tx*]_*α*_*L*_(*x*)_, where *α*_*L*_(*x*) ∈ *L*∖{0_*L*_}, with *β*(*x*, *y*) ≥ 1, we have *β*(*y*, *z*) ≥ 1 for all *z* ∈ [*Sy*]_*α*_*L*_(*y*)_ ≠ *ϕ*, where *α*_*L*_(*y*) ∈ *L*∖{0_*L*_}.

If *S* = *T* then *T* is called *β*_*ℱ*_*L*__-admissible.

**Remark 13**

It is easy to see that if (*S*, *T*) is *β*_*ℱ*_*L*__-admissible, then (*T*, *S*) is also *β*_*ℱ*_*L*__-admissible.

Next, we give a common *L*-fuzzy fixed point theorem for *β*_*F*_-admissible pair.

**Theorem**

Let (*X*, *d*) be a complete metric space, *β* : *X* × *X* → [0, *∞*), and *S*, *TL*-fuzzy mappings from *X* into *ℱ*_*L*_(*X*) satisfying the following conditions.

(a) For each *x* ∈ *X*, there exists *α*_*L*_(*x*) ∈ *L*∖{0_*L*_} such that [*Sx*]_*α*_*L*_(*x*)_, [*Tx*]_*α*_*L*_(*x*)_ are nonempty closed bounded subsets of *X* and for *x*_0_ ∈ *X*, there exists *x*_1_ ∈ [*Sx*_0_]_*α*_*L*_(*x*_0_)_ with *β*(*x*_0_, *x*_1_) ≥ 1.
(b) For all *x*, *y* ∈ *X*, we have
  (9) max⁡{β(x,y),β(y,x)}[H([Sx]αL(x),[Ty]αL(y))]  ≤a1(dx,[Sx]αL(x))+a2(dy,[Ty]αL(y))   +a3(dx,[Ty]αL(y))+a4(dy,[Sx]αL(x))+a5d(x,y),
 where *a*_1_, *a*_2_, *a*_3_, *a*_4_, and *a*_5_ are nonnegative real numbers and ∑_*i*=1_^5^ *a*_*i*_ < 1 and either *a*_1_ = *a*_2_ or *a*_3_ = *a*_4_.
(c) (*S*, *T*) is *β*_*ℱ*_*L*__-admissible pair.
(d) If {*x*_*n*_} ∈ *X*, such that *β*(*x*_*n*_, *x*_*n*+1_) ≥ 1 and *x*_*n*_ → *x*, then *β*(*x*_*n*_, *x*) ≥ 1.

Then there exists *z* ∈ *X* such that *z* ∈ [*Sz*]_*α*_*L*_(*z*)_∩[*Tz*]_*α*_*L*_(*z*)_.

**Proof**

We will prove the above result by considering the following three cases:

1. *a*_1_ + *a*_3_ + *a*_5_ = 0,
2. *a*_2_ + *a*_4_ + *a*_5_ = 0,
3. *a*_1_ + *a*_3_ + *a*_5_ ≠ 0 and *a*_2_ + *a*_4_ + *a*_5_ ≠ 0.

*Case 1*. For *x*_0_ ∈ *X* in condition (a), there exist *α*_*L*_(*x*_0_) ∈ *L*∖{0_*L*_} and *x*_1_ ∈ [*Sx*_0_]_*α*_*L*_(*x*_0_)_ such that *β*(*x*_0_, *x*_1_) ≥ 1 and also there exists *α*_*L*_(*x*_1_) ∈ *L*∖{0_*L*_} such that [*Sx*_0_]_*α*_*L*_(*x*_0_)_ and [*Tx*_1_]_*α*_*L*_(*x*_1_)_ are nonempty closed bounded subsets of *X*. From Lemma 1, we obtain that

(10) d(x1,[Tx1]αL(x1))  ≤H([Sx0]αL(x0),[Tx1]αL(x1))  ≤β(x0,x1)[H([Sx0]αL(x0),[Tx1]αL(x1))]  ≤max⁡{β(x0,x1),β(x1,x0)}    ×[H([Sx0]αL(x0),[Tx1]αL(x1))].

Now, inequality (9) implies that

(11) d(x1,[Tx1]αL(x1))  ≤a1d(x0,[Sx0]αL(x0))+a2d(x1,[Tx1]αL(x1))   +a3d(x0,[Tx1]αL(x1))   +a4d(x1,[Sx0]αL(x0))+a5d(x0,x1).

Using *a*_1_ + *a*_3_ + *a*_5_ = 0 together with the fact that *d*(*x*_1_, [*Sx*_0_]_*α*_*L*_(*x*_0_)_) = 0, we get

(12) $$\begin{array}{c} d\left(x_{1},{\left[Tx_{1}\right]}_{\alpha_{L}(x_{1})}\right)\leq a_{2}d\left(x_{1},{\left[Tx_{1}\right]}_{\alpha_{L}(x_{1})}\right). \end{array}$$

It follows that *x*_1_ ∈ [*Tx*_1_]_*α*_*L*_(*x*_1_)_, which further implies that

(13) $$\begin{array}{c} d\left(x_{1},{\left[Sx_{1}\right]}_{\alpha_{L}(x_{1})}\right)\leq H\left({\left[Tx_{1}\right]}_{\alpha_{L}(x_{1})},{\left[Sx_{1}\right]}_{\alpha_{L}(x_{1})}\right). \end{array}$$

By condition (c), for *x*_0_ ∈ *X* and *x*_1_ ∈ [*Sx*_0_]_*α*_*L*_(*x*_0_)_ such that *β*(*x*_0_, *x*_1_) ≥ 1, we have *β*(*x*_1_, *z*) ≥ 1 for all *z* ∈ [*Tx*_1_]_*α*_*L*_(*x*_1_)_. Since *x*_1_ ∈ [*Tx*_1_]_*α*_*L*_(*x*_1_)_, therefore *β*(*x*_1_, *x*_1_) ≥ 1 and hence

(14) d(x1,[Sx1]αL(x1))  ≤β(x1,x1)[H([Sx1]αL(x1),[Tx1]αL(x1))].

Again, inequality (9) implies that

(15) d(x1,[Sx1]αL(x1))  ≤a1d(x1,[Sx1]αL(x1))+a2d(x1,[Tx1]αL(x1))   +a3d(x1,[Tx1]αL(x1))   +a4d(x1,[Sx1]αL(x1))+a5d(x1,x1).

Since *a*_1_ + *a*_3_ + *a*_5_ = 0 and *d*(*x*_1_, [*Tx*_1_]_*α*_*L*_(*x*_1_)_) = 0, we get

(16) $$\begin{array}{c} d\left(x_{1},{\left[Sx_{1}\right]}_{\alpha_{L}(x_{1})}\right)\leq a_{4}d\left(x_{1},{\left[Sx_{1}\right]}_{\alpha_{L}(x_{1})}\right), \end{array}$$

which implies that *x*_1_ ∈ [*Sx*_1_]_*α*_*L*_(*x*_1_)_ and hence

(17) $$\begin{array}{c} x_{1}\in {\left[Sx_{1}\right]}_{\alpha_{L}(x_{1})}\cap {\left[Tx_{1}\right]}_{\alpha_{L}(x_{1})}. \end{array}$$

*Case 2*. For *x*_0_ ∈ *X* in condition (a), there exist *α*_*L*_(*x*_0_) ∈ *L*∖{0_*L*_} and *x*_1_ ∈ [*Sx*_0_]_*α*_*L*_(*x*_0_)_ such that *β*(*x*_0_, *x*_1_) ≥ 1 and also there exists *α*_*L*_(*x*_1_) ∈ *L*∖{0_*L*_} such that [*Sx*_0_]_*α*_*L*_(*x*_0_)_ and [*Tx*_1_]_*α*_*L*_(*x*_1_)_ are nonempty closed bounded subsets of *X*. By condition (c), we have *β*(*x*_1_, *x*_2_) ≥ 1 for all *x*_2_ ∈ [*Tx*_1_]_*α*_*L*_(*x*_1_)_. From Lemma 1, we obtain that

(18) d(x2,[Sx2]αL(x2))  ≤H([Tx1]αL(x1),[Sx2]αL(x2))  ≤β(x1,x2)[H([Sx2]αL(x2),[Tx1]αL(x1))]  ≤max⁡{β(x1,x2),β(x2,x1)}    ×[H([Sx2]αL(x2),[Tx1]αL(x1))]  ≤a1d(x2,[Sx2]αL(x2))+a2d(x1,[Tx1]αL(x1))   +a3d(x2,[Tx1]αL(x1))+a4d(x1,[Sx2]αL(x2))   +a5d(x2,x1).

Using *a*_2_ + *a*_4_ + *a*_5_ = 0 together with the fact that *d*(*x*_2_, [*Tx*_1_]_*α*_*L*_(*x*_1_)_) = 0, we get

(19) $$\begin{array}{c} d\left(x_{2},{\left[Sx_{2}\right]}_{\alpha_{L}(x_{2})}\right)\leq a_{1}d\left(x_{2},{\left[Sx_{2}\right]}_{\alpha_{L}(x_{2})}\right). \end{array}$$

It follows that *x*_2_ ∈ [*Sx*_2_]_*α*_*L*_(*x*_2_)_, which further implies that

(20) $$\begin{array}{c} d\left(x_{2},{\left[Tx_{2}\right]}_{\alpha_{L}(x_{2})}\right)\leq H\left({\left[Sx_{2}\right]}_{\alpha_{L}(x_{2})},{\left[Tx_{2}\right]}_{\alpha_{L}(x_{2})}\right). \end{array}$$

By condition (c), we have *β*(*x*_2_, *x*_2_) ≥ 1, and hence

(21) d(x2,[Tx2]αL(x2))  ≤β(x2,x2)[H([Sx2]αL(x2),[Tx2]αL(x2))].

Again, inequality (9) implies that

(22) d(x2,[Tx2]αL(x2))  ≤a1d(x2,[Sx2]αL(x2))+a2d(x2,[Tx2]αL(x2))   +a3d(x2,[Tx2]αL(x2))   +a4d(x2,[Sx2]αL(x2))+a5d(x2,x2).

Since *a*_2_ + *a*_4_ + *a*_5_ = 0 and *d*(*x*_2_, [*Sx*_2_]_*α*_*L*_(*x*_2_)_) = 0, we get

(23) $$\begin{array}{c} d\left(x_{2},{\left[Tx_{2}\right]}_{\alpha_{L}(x_{2})}\right)\leq a_{3}d\left(x_{2},{\left[Tx_{2}\right]}_{\alpha_{L}(x_{2})}\right), \end{array}$$

which implies that *x*_2_ ∈ [*Tx*_2_]_*α*_*L*_(*x*_2_)_ and hence

(24) $$\begin{array}{c} x_{2}\in {\left[Sx_{2}\right]}_{\alpha_{L}(x_{2})}\cap {\left[Tx_{2}\right]}_{\alpha_{L}(x_{2})}. \end{array}$$

*Case 3*. Let *λ* = ((*a*_1_ + *a*_3_ + *a*_5_)/(1 − *a*_2_ − *a*_3_)) and *μ* = ((*a*_2_ + *a*_4_ + *a*_5_)/(1 − *a*_1_ − *a*_4_)). Next, we show that if *a*_1_ = *a*_2_ or *a*_3_ = *a*_4_, then 0 < *λμ* < 1.

If *a*_3_ = *a*_4_, then *λ*, *μ* < 1 and so 0 < *λμ* < 1. Now if *a*_1_ = *a*_2_, then

(25) 0<λμ=(a1+a3+a51−a2−a3)(a2+a4+a51−a1−a4)=(a1+a3+a51−a1−a3)(a1+a4+a51−a1−a4)=(a1+a3+a51−a1−a4)(a1+a4+a51−a1−a3)<1.

By condition (a), for *x*_1_ ∈ *X*, there exists *α*_*L*_(*x*_1_) ∈ *L*∖{0_*L*_} such that [*Tx*_1_]_*α*_*L*_(*x*_1_)_ is a nonempty closed bounded subset of *X*. Since *a*_1_ + *a*_3_ + *a*_5_ > 0, by Lemma 2, there exists *x*_2_ ∈ [*Tx*_1_]_*α*_*L*_(*x*_1_)_ such that

(26) d(x1,x2)  ≤H([Sx0]αL(x0),[Tx1]αL(x1))+a1+a3+a5  ≤β(x0,x1)[H([Sx0]αL(x0),[Tx1]αL(x1))]   +a1+a3+a5  ≤max⁡{β(x0,x1),β(x1,x0)}    ×[H([Sx0]αL(x0),[Tx1]αL(x1))]+a1+a3+a5  ≤a1d(x0,[Sx0]αL(x0))+a2d(x1,[Tx1]αL(x1))   +a3d(x0,[Tx1]αL(x1))+a4d(x1,[Sx0]αL(x0))   +a5d(x0,x1)+a1+a3+a5  ≤(a1+a5)d(x0,x1)+a2d(x1,x2)   +a3d(x0,x2)+a1+a3+a5  ≤(a1+a3+a5)d(x0,x1)+(a2+a3)d(x1,x2)   +a1+a3+a5.

This implies that

(27) $$\begin{array}{c} d\left(x_{1},x_{2}\right)\leq \lambda d\left(x_{0},x_{1}\right)+\lambda . \end{array}$$

By the same argument, for *x*_2_ ∈ *X*, there exists *α*_*L*_(*x*_2_) ∈ *L*∖{0_*L*_} such that [*Sx*_2_]_*α*_*L*_(*x*_2_)_ is a nonempty closed bounded subset of *X*. Since *a*_2_ + *a*_4_ + *a*_5_ > 0, by Lemma 2, there exists *x*_3_ ∈ [*Sx*_2_]_*α*_*L*_(*x*_2_)_ such that

(28) $$\begin{array}{c} d\left(x_{2},x_{3}\right)\leq H\left({\left[Tx_{1}\right]}_{\alpha_{L}(x_{1})},{\left[Sx_{2}\right]}_{\alpha_{L}(x_{2})}\right)+\lambda \left(a_{2}+a_{4}+a_{5}\right). \end{array}$$

By condition (c), for *x*_0_ ∈ *X* and *x*_1_ ∈ [*Sx*_0_]_*α*_*L*_(*x*_0_)_ such that *β*(*x*_0_, *x*_1_) ≥ 1, we have *β*(*x*_1_, *x*_2_) ≥ 1 for *x*_2_ ∈ [*Tx*_1_]_*α*_*L*_(*x*_1_)_. So we have

(29) d(x2,x3)  ≤H([Tx1]αL(x1),[Sx2]αL(x2))+λ(a2+a4+a5)  =H([Sx2]αL(x2),[Tx1]αL(x1))+λ(a2+a4+a5)  ≤β(x1,x2)[H([Sx2]αL(x2),[Tx1]αL(x1))]   +λ(a2+a4+a5)  ≤max⁡{β(x1,x2),β(x2,x1)}    ×[H([Sx2]αL(x2),[Tx1]αL(x1))]+λ(a2+a4+a5)  ≤a1d(x2,[Sx2]αL(x2))+a2d(x1,[Tx1]αL(x1))   +a3d(x2,[Tx1]αL(x1))+a4d(x1,[Sx2]αL(x2))   +a5d(x2,x1)+λ(a2+a4+a5)  ≤a1d(x2,x3)+(a2+a5)d(x1,x2)   +a4d(x1,x3)+λ(a2+a4+a5)  ≤(a2+a4+a5)d(x1,x2)   +(a1+a4)d(x2,x3)+λ(a2+a4+a5).

This implies that

(30) $$\begin{array}{c} d\left(x_{2},x_{3}\right)\leq \mu d\left(x_{1},x_{2}\right)+\lambda \mu . \end{array}$$

By repeating the above process, for *x*_3_ ∈ *X*, there exists *α*_*L*_(*x*_3_) ∈ *L*∖{0_*L*_} such that [*Tx*_3_]_*α*_*L*_(*x*_3_)_ is a nonempty closed bounded subset of *X*. From Lemma 2, there exists *x*_4_ ∈ [*Tx*_3_]_*α*_*L*_(*x*_3_)_ such that

(31) $$\begin{array}{c} d\left(x_{3},x_{4}\right)\leq H\left({\left[Sx_{2}\right]}_{\alpha_{L}(x_{2})},{\left[Tx_{3}\right]}_{\alpha_{L}(x_{3})}\right)+\lambda \mu \left(a_{1}+a_{3}+a_{5}\right). \end{array}$$

By condition (c), for *x*_1_ ∈ *X* and *x*_2_ ∈ [*Tx*_1_]_*α*_*L*_(*x*_1_)_ such that *β*(*x*_1_, *x*_2_) ≥ 1, we have *β*(*x*_2_, *x*_3_) ≥ 1 for *x*_3_ ∈ [*Sx*_2_]_*α*_*L*_(*x*_2_)_. So we have

(32) d(x3,x4)  ≤H([Sx2]αL(x2),[Tx3]αL(x3))   +λμ(a1+a3+a5)  ≤β(x2,x3)[H([Sx2]αL(x2),[Tx3]αL(x3))]   +λμ(a1+a3+a5)  ≤max⁡{β(x2,x3),β(x3,x2)}    ×[H([Sx2]αL(x2),[Tx3]αL(x3))]    +λμ(a1+a3+a5)  ≤a1d(x2,[Sx2]αL(x2))+a2d(x3,[Tx3]αL(x3))   +a3d(x2,[Tx3]αL(x3))+a4d(x3,[Sx2]αL(x2))   +a5d(x2,x3)+λμ(a1+a3+a5)  ≤(a1+a5)d(x2,x3)+a2d(x3,x4)   +a3d(x2,x4)+λμ(a1+a3+a5)  ≤(a1+a3+a5)d(x2,x3)   +(a2+a3)d(x3,x4)+λμ(a1+a3+a5).

This implies that

(33) $$\begin{array}{c} d\left(x_{3},x_{4}\right)\leq \lambda d\left(x_{2},x_{3}\right)+\lambda \left(\lambda \mu \right). \end{array}$$

By induction, we produce a sequence {*x*_*n*_} in *X* such that

(34) $$\begin{array}{c} x_{2k+1}\in {\left[Sx_{2k}\right]}_{\alpha_{L}(x_{2k})},\\ x_{2k+2}\in {\left[Tx_{2k+1}\right]}_{\alpha_{L}(x_{2k+1})},\;k=0,1,2,\ldots ,\\ \beta \left(x_{n-1},x_{n}\right)\geq 1,\;\forall n\in \mathbb{N}. \end{array}$$

Now, we have

(35) d(x2k+1,x2k+2)  ≤H([Sx2k]αL(x2k),[Tx2k+1]αL(x2k+1))   +(λμ)k(a1+a3+a5)  ≤β(x2k,x2k+1)   ×[H([Sx2k]αL(x2k),[Tx2k+1]αL(x2k+1))]   +(λμ)k(a1+a3+a5)  ≤max⁡{β(x2k,x2k+1),β(x2k+1,x2k)}    ×[H([Sx2k]αL(x2k),[Tx2k+1]αL(x2k+1))]    +(λμ)k(a1+a3+a5)  ≤a1d(x2k,[Sx2k]αL(x2k))   +a2d(x2k+1,[Tx2k+1]αL(x2k+1))   +a3d(x2k,[Tx2k+1]αL(x2k+1))   +a4d(x2k+1,[Sx2k]αL(x2k))+a5d(x2k,x2k+1)   +(λμ)k(a1+a3+a5)  ≤(a1+a5)d(x2k,x2k+1)   +a2d(x2k+1,x2k+2)+a3d(x2k,x2k+2)   +(λμ)k(a1+a3+a5)  ≤(a1+a3+a5)d(x2k,x2k+1)   +(a2+a3)d(x2k+1,x2k+2)+(λμ)k(a1+a3+a5).

This implies that

(36) $$\begin{array}{c} d\left(x_{2k+1},x_{2k+2}\right)\leq \lambda d\left(x_{2k},x_{2k+1}\right)+\lambda {\left(\lambda \mu \right)}^{k}. \end{array}$$

Similarly,

(37) d(x2k+2,x2k+3)  ≤H([Sx2k+2]αL(x2k+2),[Tx2k+1]αL(x2k+1))   +(λμ)kλ(a2+a4+a5)  ≤β(x2k+1,x2k+2)   ×[H([Sx2k+2]αL(x2k+2),[Tx2k+1]αL(x2k+1))]   +(λμ)kλ(a2+a4+a5)  ≤max⁡{β(x2k+1,x2k+2),β(x2k+2,x2k+1)}    ×[H([Sx2k+2]αL(x2k+2),[Tx2k+1]αL(x2k+1))]    +(λμ)kλ(a2+a4+a5)  ≤a1d(x2k+2,[Sx2k+2]αL(x2k+2))   +a2d(x2k+1,[Tx2k+1]αL(x2k+1))   +a3d(x2k+2,[Tx2k+1]αL(x2k+1))   +a4d(x2k+1,[Sx2k+2]αL(x2k+2))   +a5d(x2k+2,x2k+1)+(λμ)kλ(a2+a4+a5)  ≤a1d(x2k+2,x2k+3)   +(a2+a5)d(x2k+1,x2k+2)   +a4d(x2k+1,x2k+3)+(λμ)kλ(a2+a4+a5)  ≤(a2+a4+a5)d(x2k+1,x2k+2)   +(a1+a4)d(x2k+2,x2k+3)   +(λμ)kλ(a2+a4+a5).

This implies that

(38) $$\begin{array}{c} d\left(x_{2k+2},x_{2k+3}\right)\leq \mu d\left(x_{2k+1},x_{2k+2}\right)+{\left(\lambda \mu \right)}^{k+1}. \end{array}$$

From (36) and (38), it follows that, for each *k* = 0,1, 2,…,

(39) d(x2k+1,x2k+2)  ≤λd(x2k,x2k+1)+λ(λμ)k  ≤λ[μd(x2k−1,x2k)+(λμ)k]+λ(λμ)k  =(λμ)d(x2k−1,x2k)+2λ(λμ)k  ≤(λμ)[λd(x2k−2,x2k−1)+λ(λμ)k−1]+2λ(λμ)k  =(λμ)λd(x2k−2,x2k−1)+3λ(λμ)k   ⋮  ≤λ(λμ)kd(x0,x1)+(2k+1)λ(λμ)k,d(x2k+2,x2k+3)  ≤μd(x2k+1,x2k+2)+(λμ)k+1   ⋮  ≤(λμ)k+1d(x0,x1)+(2k+2)(λμ)k+1.

Then for *m* < *n*, we have

(40) d(x2m+1,x2n+1)≤d(x2m+1,x2m+2)+d(x2m+2,x2m+3) +d(x2m+3,x2m+4)+⋯+d(x2n,x2n+1)≤[λ∑i=mn−1(λμ)i+∑i=m+1n(λμ)i]d(x0,x1) +λ∑i=mn−1(2i+1)(λμ)i+∑i=m+1n2i(λμ)i.

Similarly, we obtain that

(41) d(x2m,x2n+1)≤[∑i=mn(λμ)i+λ∑i=mn−1(λμ)i]d(x0,x1) +∑i=mn2i(λμ)i+λ∑i=mn−1(2i+1)(λμ)i,d(x2m,x2n)≤[∑i=mn−1(λμ)i+λ∑i=mn−1(λμ)i]d(x0,x1) +∑i=mn−12i(λμ)i+λ∑i=mn−1(2i+1)(λμ)i,d(x2m+1,x2n)≤[λ∑i=mn−1(λμ)i+∑i=m+1n−1(λμ)i]d(x0,x1) +λ∑i=mn−1(2i+1)(λμ)i+∑i=m+1n−12i(λμ)i.

Since 0 < *λμ* < 1, so by Cauchy's root test, we get ∑(2*i* + 1)(*λμ*)^*i*^ and ∑2*i*(*λμ*)^*i*^ are convergent series. Therefore, {*x*_*n*_} is a Cauchy sequence in *X*. Now, from the completeness of *X*, there exists *z* ∈ *X* such that *x*_*n*_ → *z* as *n* → *∞*. By condition (d), we have *β*(*x*_*n*−1_, *z*) ≥ 1 for all *n* ∈ *ℕ*. Now, we have

(42) d(x2n,[Sz]α(z))  ≤H([Tx2n−1]αL(x2n−1),[Sz]α(z))  =H([Sz]α(z),[Tx2n−1]αL(x2n−1))  ≤β(x2n−1,z)H([Sz]α(z),[Tx2n−1]αL(x2n−1))  ≤max⁡{β(x2n−1,z),β(z,x2n−1)}    ×H([Sz]α(z),[Tx2n−1]αL(x2n−1))  ≤a1d(z,[Sz]αL(z))   +a2d(x2n−1,[Tx2n−1]αL(x2n−1))   +a3d(z,[Tx2n−1]αL(x2n−1))   +a4d(x2n−1,[Sz]αL(z))+a5d(z,x2n−1)  ≤a1d(z,[Sz]αL(z))+a2d(x2n−1,x2n)+a3d(z,x2n)   +a4d(x2n−1,[Sz]αL(z))+a5d(z,x2n−1).

Since

(43) $$\begin{array}{c} d\left(z,{\left[Sz\right]}_{\alpha_{L}(z)}\right)\leq d\left(z,x_{2n}\right)+d\left(x_{2n},{\left[Sz\right]}_{\alpha_{L}(z)}\right), \end{array}$$

so we get

(44) d(z,[Sz]αL(z))  ≤d(z,x2n)+a1d(z,[Sz]αL(z))   +a2d(x2n−1,x2n)+a3d(z,x2n)   +a4d(x2n−1,[Sz]αL(z))+a5d(z,x2n−1)  ≤(1+a3)d(z,x2n)   +(a4+a5)d(z,x2n−1)+a2d(x2n−1,x2n)   +(a1+a4)d(z,[Sz]αL(z)).

This implies that

(45) d(z,[Sz]αL(z))  ≤(1+a31−a1−a4)d(z,x2n)   +(a4+a51−a1−a4)d(z,x2n−1)   +(a21−a1−a4)d(x2n−1,x2n).

Letting *n* → *∞*, we have *d*(*z*, [*Sz*]_*α*_*L*_(*z*)_) = 0. It implies that *z* ∈ [*Sz*]_*α*_*L*_(*z*)_. Similarly, by using

(46) $$\begin{array}{c} d\left(z,{\left[Tz\right]}_{\alpha_{L}(z)}\right)\leq d\left(z,x_{2n+1}\right)+d\left(x_{2n+1},{\left[Tz\right]}_{\alpha_{L}(z)}\right), \end{array}$$

we can show that *z* ∈ [*Tz*]_*α*_*L*_(*z*)_. Therefore, *z* ∈ [*Sz*]_*α*_*L*_(*z*)_∩[*Tz*]_*α*_*L*_(*z*)_. This completes the proof.

Next, we give an example to support the validity of our result.

**Example**

Let *X* = [0,1], *d*(*x*, *y*) = |*x* − *y*|, whenever *x*, *y* ∈ *X*; then (*X*, *d*) is a complete metric space. Let *L* = {*δ*, *ω*, *τ*, *κ*} with *δ* ≾_*L*_ *ω* ≾_*L*_ *κ*, *δ* ≾_*L*_ *τ* ≾_*L*_ *κ*, *ω* and *τ* are not comparable; then (*L*, ≾_*L*_) is a complete distributive lattice. Define a pair of mappings *S*, *T* : *X* → *ℱ*_*L*_(*X*) as follows:

(47) $$\begin{array}{c} S\left(x\right)\left(t\right)=\{\begin{array}{cc} \kappa , & \text{if}\;\;0\leq t\leq \frac{x}{6},\\ \omega , & \text{if}\;\;\frac{x}{6}<t\leq \frac{x}{3},\\ \tau , & \text{if}\;\;\frac{x}{3}<t\leq \frac{x}{2},\\ \delta , & \text{if}\;\;\frac{x}{2}<t\leq 1, \end{array}\\ T\left(x\right)\left(t\right)=\{\begin{array}{cc} \kappa , & \text{if}\;\;0\leq t\leq \frac{x}{12},\\ \delta , & \text{if}\;\;\frac{x}{12}<t\leq \frac{x}{8},\\ \omega , & \text{if}\;\;\frac{x}{8}<t\leq \frac{x}{4},\\ \tau , & \text{if}\;\;\frac{x}{4}<t\leq 1. \end{array} \end{array}$$

Define *β* : *X* × *X* → [0, *∞*) as follows:

(48) $$\begin{array}{c} \beta \left(x,y\right)=\{\begin{array}{cc} \frac{1}{\left|x-y\right|}, & x\neq y,\\ 1, & x=y. \end{array} \end{array}$$

For all *x* ∈ *X*, there exists *α*_*L*_(*x*) = *κ*, such that

(49) $$\begin{array}{c} {\left[Sx\right]}_{\kappa }=\left[0,\frac{x}{6}\right],\;\;{\left[Tx\right]}_{\kappa }=\left[0,\frac{x}{12}\right], \end{array}$$

and all conditions of the above theorem are satisfied. Hence, there exists 0 ∈ *X*, such that 0 ∈ [*S*0]_*α*_*L*_(0)_∩[*T*0]_*α*_*L*_(0)_.

**Corollary**

Let (*X*, *d*) be a complete metric space, *β* : *X* × *X* → [0, *∞*), and *S*, *T* fuzzy mappings from *X* into *ℱ*(*X*) satisfying the following conditions.

(a) For each *x* ∈ *X*, there exists *α*(*x*) ∈ (0,1] such that [*Sx*]_*α*(*x*)_, [*Tx*]_*α*(*x*)_ are nonempty closed bounded subsets of *X* and for *x*_0_ ∈ *X*, there exists *x*_1_ ∈ [*Sx*_0_]_*α*(*x*_0_)_ with *β*(*x*_0_, *x*_1_) ≥ 1.
(b) For all *x*, *y* ∈ *X*, we have
  (50) max⁡{β(x,y),β(y,x)}[H([Sx]α(x),[Ty]α(y))]  ≤a1(dx,[Sx]α(x))+a2(dy,[Ty]α(y))   +a3(dx,[Ty]α(y))   +a4(dy,[Sx]α(x))+a5d(x,y),
 where *a*_1_, *a*_2_, *a*_3_, *a*_4_, and *a*_5_ are nonnegative real numbers and ∑_*i*=1_^5^ *a*_*i*_ < 1 and either *a*_1_ = *a*_2_ or *a*_3_ = *a*_4_.
(c) (*S*, *T*) is *β*_*ℱ*_-admissible pair.
(d) If {*x*_*n*_} ∈ *X*, such that *β*(*x*_*n*_, *x*_*n*+1_) ≥ 1 and *x*_*n*_ → *x* then *β*(*x*_*n*_, *x*) ≥ 1.

Then there exists *z* ∈ *X* such that *z* ∈ [*Sz*]_*α*(*z*)_∩[*Tz*]_*α*(*z*)_.

**Proof**

Consider an *L*-fuzzy mapping *A* : *X* → *ℱ*_*L*_(*X*) defined by

(51) $$\begin{array}{c} Ax=\chi_{L_{Tx}}. \end{array}$$

Then for *α*_*L*_ ∈ *L*∖{0_*L*_}, we have

(52) $$\begin{array}{c} {\left[Ax\right]}_{\alpha_{L}}=Tx. \end{array}$$

Hence by Theorem 14, we follow the result.

If we set *β*(*x*, *y*) = 1 for all *x*, *y* ∈ *X* in Corollary 16, we get the following result.

**Corollary 17 (see [26])**

Let (*X*, *d*) be a complete metric space and *S*, *T* fuzzy mappings from *X* into *ℱ*(*X*) satisfying the following conditions:

(a) for each *x* ∈ *X*, there exists *α*(*x*) ∈ (0,1] such that [*Sx*]_*α*(*x*)_, [*Tx*]_*α*(*x*)_ are nonempty closed bounded subsets of *X*;
(b) for all *x*, *y* ∈ *X*, we have
  (53) H([Sx]α(x),[Ty]α(y)) ≤a1d(x,[Sx]α(x))+a2d(y,[Ty]α(y))  +a3d(x,[Ty]α(y))+a4d(y,[Sx]α(x))  +a5d(x,y),
 where *a*_1_, *a*_2_, *a*_3_, *a*_4_, and *a*_5_ are nonnegative real numbers and ∑_*i*=1_^5^ *a*_*i*_ < 1 and either *a*_1_ = *a*_2_ or *a*_3_ = *a*_4_.

Then there exists *z* ∈ *X* such that *z* ∈ [*Sz*]_*α*(*z*)_∩[*Tz*]_*α*(*z*)_.
